# Monkeys overharvest shellfish

**DOI:** 10.7554/eLife.30865

**Published:** 2017-09-13

**Authors:** George H Perry, Brian F Codding

**Affiliations:** 1 Department of AnthropologyPennsylvania State UniversityUniversity ParkUnited States; 2 Department of BiologyPennsylvania State UniversityUniversity ParkUnited States; 3 Huck Institutes of the Life SciencesPennsylvania State UniversityUniversity ParkUnited States; 4 Department of AnthropologyUniversity of UtahSalt Lake CityUnited States; 5 Global Change and Sustainability CenterUniversity of UtahSalt Lake CityUnited States

**Keywords:** Macaca fascicularis, stone tool use, shellfish, Thailand, Other

## Abstract

The use of stone tools by macaques in Thailand has reduced the size and population density of coastal shellfish; previously it was thought that tool-assisted overharvesting effects resulted uniquely from human activity.

**Related research article** Luncz LV, Tan A, Haslam M, Kulik L, Proffitt T, Malaivijitnond S, Gumert M. 2017. Resource depletion through primate stone technology. *eLife* **6**:e23647. doi: 10.7554/eLife.23647

The use of tools can enable animals to expand their dietary options by making it easier to acquire food that is structurally protected (by spines or shells, for example) or hidden. Tool use is also generally associated with increases in the efficiency of food harvesting. Some of the hunting and fishing tools used by humans are so efficient that they have played roles in the local and even global extinction of some species of prey (Dirzo et al., 2014; Boivin et al., 2016).

Human tool-assisted harvesting can impact the biology of a prey species in several ways (Fenberg and Roy, 2008). Overharvesting, especially where larger individuals are preferentially targeted, can lead to the members of the harvested population being younger and smaller on average compared to non-harvested populations, especially for species (like many shellfish) that continue to grow throughout life. Human size-selective harvesting or trophy hunting can also result in evolutionary change, with genetic variants that confer smaller body or feature size becoming increasingly common in the affected prey population.

Biologists have documented many examples of these effects being driven by human harvesting pressures, and an archaeological record of these processes also extends at least 50,000 years into the past (Sullivan et al., 2017). This record is especially extensive for shellfish, as size changes over time can be quantified from shells discarded in the different layers of prehistoric trash mounds, or middens.

Before Jane Goodall observed chimpanzees using modified twigs to ‘fish’ for termites (Goodall, 1964), it was widely thought that tool use was a uniquely human trait. Behavioral scientists have since gathered detailed evidence of habitual tool use by multiple other primate species, including orang-utans, macaques, and capuchin monkeys (Figure 1), and also by non-primates including crows and dolphins (Koops et al., 2014; Rutz et al., 2016). Now, in eLife, Lydia Luncz and colleagues provide the first report of a tool-assisted overharvesting process driven by non-humans (Luncz et al., 2017).

**Figure 1.**
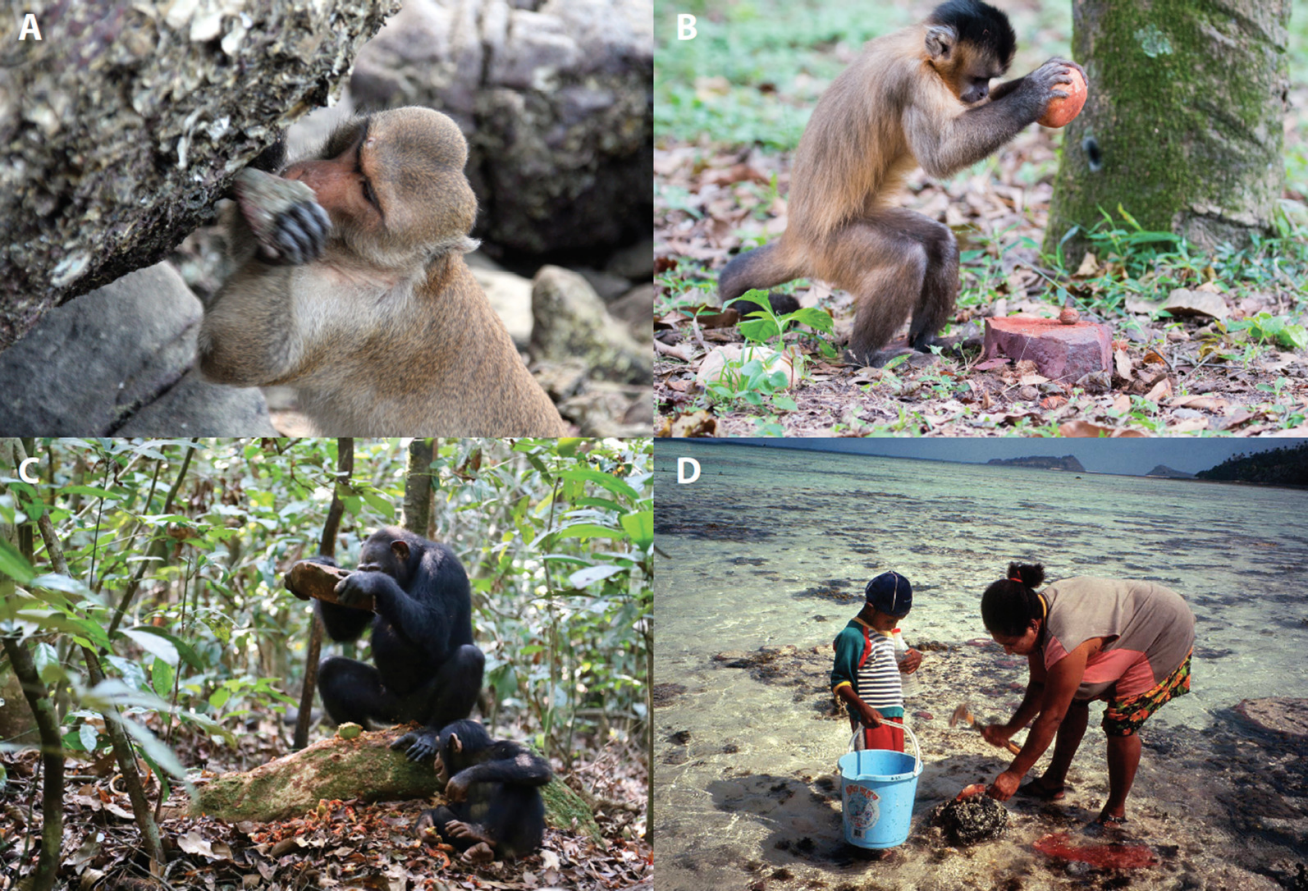
Using tools to acquire and process food. (**A**) Long-tailed macaque (*M. fascicularis*) on Koram Island in the Khao Sam Roi Yot National Park, Thailand. Luncz et al. report that macaques on this island are using stone tools to overharvest local shellfish populations. *Photograph: Amanda Tan*. (**B**) Juvenile capuchin monkey (*Sapajus* sp.) in the Tietê Ecological Park in São Paulo, Brazil, cracking a nut with a stone tool. *Photograph: Tiago Falótico*. (**C**) An adult chimpanzee (*Pan troglodytes*) uses a stone tool, as a juvenile chimpanzee looks on, in the Taï National Park, Côte d'Ivoire. *Photograph: Liran Samuni/Taï Chimpanzee Project*. (**D**) Meriam Islander Sonia Passi and her son JJ Passi, Mer Island, Torres Strait, Australia, collecting and processing spider conch, or *asor*, in Meriam Mir (*Lambis lambis*). *Photograph: Douglas Bird*.

Long-tailed macaques (*Macaca fascicularis*) on two islands in the Khao Sam Roi Yot National Park in Thailand use stone tools to break open and access the meat of coastal oysters and other shellfish (Tan, 2017). Taking advantage of a natural experiment, Luncz et al. examined differences in shellfish sizes and stone tool use between Koram Island, where 26 macaques used stone tools to process shellfish along a shoreline length of 1551 m (equivalent to 55.4 m of shoreline per individual macaque), and nearby NomSao Island, where only four individuals harvested shellfish along a 653 m shoreline (163.3 m per individual). Thus, although ecological conditions for shellfish on the two islands are otherwise similar, the shellfish on Koram Island are likely harvested approximately three times more intensively than those on NomSao Island.

Luncz et al. – who are based at the University of Oxford, Nanyang Technical University, the Max Planck Institute for Evolutionary Anthropology, Chulalongkorn University, and the National Primate Research Center of Thailand – discovered that multiple prey species of shellfish on Koram Island had significantly lower population densities and smaller average body sizes than those on NomSao Island. For example, the average Koram rock oyster was about 60% the size of its counterpart on NomSao. The size differences appear to reflect life history alterations rather than evolutionary genetic processes, because while shellfish at similar stages of maturity were similar in size on both islands, there were proportionally fewer sexually mature individuals on Koram.

Fascinatingly, Koram macaques also selected significantly smaller stones to process their (commensurately smaller) shellfish, despite the fact that small stones were relatively less abundant on this island than on NomSao. This result neatly illustrates how technological innovations can themselves be driven by overexploitation. That is, relatively high intensity foraging by tool-using macaques likely led to reductions in the average sizes of local shellfish, necessitating the use of smaller tools (which in turn could further drive down shellfish sizes, and so on). A similar feedback loop was previously proposed to drive technological innovation for human hunter-gatherers (e.g., Morgan, 2015).

We now know that tool-assisted overharvesting is not unique to human-prey interactions. An important direction for future research will be to determine whether the foraging activities of other long-tailed macaque populations and other tool-using non-human species drive similar ecological changes. This study may also encourage investigation into potential longer-term, evolutionary effects on harvested prey in response to the tool-assisted foraging behavior of non-human species. Archaeological records of tool use for multiple non-human primate species that extend up to 4,300 years into the past (Mercader et al., 2007; Haslam et al., 2016) present excellent starting points for such a search.
